# Understanding men’s attitudes toward the justification of wife-beating in Tanzania: insights from the 2022 Demographic and Health Survey

**DOI:** 10.1080/16549716.2026.2656563

**Published:** 2026-04-13

**Authors:** Elihuruma Eliufoo Stephano, Tegemea Patrick Mwalingo, Azan Abubakar Nyundo, Mtoro J. Mtoro

**Affiliations:** a Department of Clinical Nursing, School of Nursing and Public Health, University of Dodoma, Dodoma, Tanzania; b Clinical Nursing Teaching and Research Section, the Second Xiangya Hospital, Central South University, Changsha, Hunan, China; c Department of Psychiatry and Mental Health, School of Medicine and Dentistry, The University of Dodoma, Dodoma, Tanzania; d TILAM International, Dar es Salaam, Tanzania

**Keywords:** Attitude, wife-beating, justification, men, Tanzania

## Abstract

**Background:**

Male justification of wife-beating refers to a man’s belief that it is acceptable or legitimate to physically discipline or assault his wife under certain circumstances. Research has predominantly focused on women, leaving a critical gap in understanding men’s attitudes and beliefs surrounding violence against women.

**Objective:**

This study aims to address this gap by examining the factors associated with male attitudes toward the justification of wife-beating in Tanzania.

**Methods:**

This study employed an analytical cross-sectional design using secondary data from the 2022 Tanzania Demographic and Health Survey, which included 5,763 ever-married men. A modified Poisson regression model identified factors, and results were presented as weighted prevalence ratios (PR) with 95% confidence intervals (CI).

**Results:**

The overall prevalence of male justification of wife-beating was 30.4% (95% CI: 28.1–32.9). Younger men, aged 15–24 years (Adjusted Prevalence Ratio [APR] = 1.62, 95% CI: 1.27–2.07), those aged 25–34 years (APR = 1.16, 95% CI: 1.01–1.34), men who consume alcohol (APR = 1.34, 95% CI: 1.17–1.53), those who were cohabiting (APR = 1.31, 95% CI: 1.10–1.55) and in men residing in the western, northern, central, southern, and lake zones of mainland Tanzania exhibited significantly higher prevalence of wife-beating justification. Conversely, men with secondary/higher education (APR = 0.73, 95% CI: 0.57–0.94) and in the middle wealth quantile (APR = 0.81, 95% CI: 0.66–0.99) were less likely to justify wife-beating.

**Conclusion:**

This study reveals a notable prevalence of male justification of wife-beating in Tanzania, with significant associations identified between key demographic and behavioral factors. These findings underscore the need for multi-pronged strategies that engage men, address socio-cultural norms, and harmful attitudes.

## Paper context

- **Main findings:** The study identified a significant prevalence of male justification of intimate partner violence in Tanzania, with younger age, alcohol consumption, cohabitation, and residence in specific geographical zones being associated with higher justification, while higher education and middle wealth were associated with lower justification.
- **Added knowledge:** This research contributes by specifically addressing the under-researched area of men’s attitudes toward intimate partner violence justification within a Tanzanian context, utilizing recent national data to identify key demographic and behavioral factors influencing these views.
- **Global health impact for policy and action:** The findings provide actionable insights for designing targeted interventions and policies that engage men and boys to challenge harmful gender norms and reduce intimate partner violence justification, contributing to global efforts to prevent gender-based violence.

## Background

Violence against women is a global crisis affecting a significant portion of the female population, whereby many victims experience this abuse from their intimate partners [1]. These violences represent a severe global public health and human rights concern [2], with approximately 27% of women worldwide experiencing physical and/or sexual violence from their partners during their lifetime [3]. The situation is particularly alarming in sub-Saharan Africa (SSA), where prevalence ranges from 36% to 71% in many countries, among the highest globally [3–5]. These elevated rates often correlate with patriarchal social structures, gender inequality, and cultural norms that normalize violence as a form of control within intimate relationships [5].

Within this context, understanding male justifications for wife-beating is critical, as these beliefs serve as cognitive mechanisms that enable and maintain abusive behaviors across diverse settings [6]. Several common risk factors contribute to male justifications for wife-beating, including male interpersonal aggression, substance abuse, childhood exposure to violence, and gendered attitudes toward aggression [7]. Additional factors that increase the likelihood of violent behavior include socioeconomic pressures, cultural conditioning, being in teen violent relationships, and access to firearms [8]. Globally, women are more likely to be victims, and common justifications for wife-beating from males include self-defense, being provoked, and anger issues. Most male perpetrators narrate wife-beating as an unintentional and unplanned action [9].

In Tanzania, male justification of wife-beating remains pervasive, with previous studies reporting that approximately 12%–61% of ever-married women have experienced physical or sexual violence from their partners [10,11]. This high prevalence interacts with traditional gender norms, socioeconomic factors, and limited support systems for survivors [3]. Studies have identified common male justification of wife-beating among Tanzanian, including perceived female transgressions of traditional gender roles (neglecting children, arguing with husband), suspected infidelity, financial disputes, women’s refusal of sexual advances, and challenging male authority in household decision-making [10,12,13]. These justifications often reflect deeply embedded cultural beliefs about male dominance and female subordination that transcend educational and socioeconomic boundaries [13].

Tanzania’s legal framework has evolved to address violence through several key measures, including the Law of Marriage Act, the Sexual Offences Special Provisions Act, and the Anti-Trafficking in Persons Act [14]. Additionally, Tanzania ratified the Convention on the Elimination of All Forms of Discrimination Against Women (CEDAW) and developed a National Plan of Action for the Prevention and Eradication of Violence Against Women and Children [15]. However, implementation remains inconsistent, with significant gaps between legal protection and practical enforcement, particularly in rural areas where traditional authorities often supersede formal legal systems [16]. This dissonance between legal frameworks and lived experiences creates an environment where male justification of wife-beating persists despite policy reforms.

Despite these legal provisions, previous research in Tanzania using the Tanzania Demographic and Health Survey (TDHS) data reveals a concerning pattern in which a substantial proportion of Tanzanian men continues to justify wife-beating under certain circumstances [16,17]. This acceptance exists alongside high actual wife-beating prevalence and creates a cycle where violence is simultaneously perpetrated, experienced, and normalized. These studies further indicate significant regional variations in attitudes toward wife-beating, with higher acceptance estimates in rural areas, among less educated populations, and in specific regions such as the Lake Zone and the Western Zone [16–18]. These variations suggest that wife-beating justification is influenced by complex intersections of geographic, cultural, and socioeconomic factors that require further understanding, underscoring the need for this secondary analysis using the TDHS.

A comprehensive study examining male justification of wife-beating in Tanzania is urgently needed to address knowledge gaps in existing research. While previous studies have documented violence prevalence and general attitudes, fewer have specifically explored the justifications used by men to rationalize violence [3,10,11,16–19]. In Tanzania, there is a scarcity of studies assessing male justification for wife-beating, particularly using nationally representative data. The TDHS offers a unique opportunity to examine these justifications across different demographic segments, enabling the development of interventions that address the root beliefs sustaining violent behaviors. Understanding male perspectives is essential because men are primarily the perpetrators [9], and sustainable violence reduction requires engaging them as agents of change. Such research would inform more effective prevention strategies that target specific reported justifications, helping Tanzania progress toward its commitments to gender equality and violence reduction. Therefore, this study aims to assess male justification of wife-beating by using a secondary analysis of the 2022 TDHS.

## Methods

### Study design and setting

This study employed an analytical cross-sectional design using secondary data from the 2022 TDHS. The data used in this study were the 2022 TDHS data. Details on DHS have been described elsewhere [20]. The TDHS was conducted in the United Republic of Tanzania from 24 February to 21 July 2022. Tanzania is a country in East Africa with approximately 62 million people, according to the 2022 census, of whom half are men [21].

## Participant and sampling

The detailed TDHS methodology has been explained elsewhere [20]. In summary, the 2022 TDHS uses a stratified two-stage sampling strategy. The first stage involved the selection of clusters consisting of census enumeration areas (EAs) delineated from the 2022 national population census. First, 629 EAs (211 urban and 418 rural) were selected with probability proportional to size (PPS) within each stratum. During the household listing phase, field staff mapped these clusters and recorded all residential addresses to create a secondary sampling frame. From this, 26 households were systematically chosen per cluster. The final sample targeted men aged 15–49, resulting in a total of 5,763 participants. In DHS methodology, participants aged 15–17  years are considered part of the reproductive-age population and are routinely included in modules assessing attitudes, reproductive health, and related outcomes. For this analysis, we analyzed data from 3,246 weighted male participants, aged ≥ 15 years, after excluding never-married men for accurate wife-beating attitude measurement.

## Variable measurements

### Dependent variable

The outcome variable for this study was male attitude toward wife-beating. This variable was derived from responses to five questions within the 2022 TDHS, each assessing whether men believed specific scenarios justified wife-beating. A binary variable was created, where ‘justification’ was defined as a ‘yes’ response to at least one of the following scenarios in Table 1. This composite variable reflects the justification of wife-beating against women under specific circumstances, as measured by the TDHS.

**Table 1.** Description of male justification toward wife-beating.

Question	Scenarios
Beating justified if;	Wife goes out without telling husband
	Wife neglects children
	Wife argues with husband
	Wife refuses to have sex with husband
	Wife burns food

### Independent variables

Independent variables were selected based on the available data and literature [3,10,11,16–19]; age in years (15–24, 25–34, or 35–49), education level (no formal education, primary education, or secondary/higher), marital status (married, cohabiting, or separated/divorced), literacy (literate; men were considered literate if they could read aloud all or part of a sentence shown to them and illiterate if otherwise) [22]. Working status (working or not working), own a mobile phone (yes or no), internet use (ever use or never use), media exposure (yes as listening to the radio, reading the newspaper, or watching Television less than once a week or at least once a week or no if otherwise), number of living children (none, 1–3, or ≥4), alcohol consumption (yes or no), household members (≤5 or ≥6), spouse current pregnant (yes or no), wealth index (poor, middle, or rich), sex of household head (male or female), residence (urban or rural), and geographical zones (western, northern, central, southern, lake, eastern, and Zanzibar).

## Data management and analysis

To address the complex survey design of the 2022 TDHS, sample weights, primary sampling units (clusters), and strata were used to adjust for clustering and potential sampling biases. Data cleaning, coding, and analysis were performed using Stata 18.5 (STATA Corp., College Station, TX) with the ‘svy’ command used in all statistical analyses. Descriptive statistics, including means, standard deviations (SD), medians (interquartile ranges), and frequencies with proportions, were used to summarize the continuous and categorical variables. The prevalence of men’s justification of wife-beating was calculated as the proportion of men reporting justification across at least one domain. Pearson’s chi-square tests were employed to compare wife-beating justification across sociodemographic characteristics.

We used a Modified Poisson regression model with a robust variance estimator as the odds ratio estimated from logistic regression might overestimate the association for non-rare outcomes (>10%). Univariate analysis was performed by fitting each explanatory variable against the response variable to provide a crude prevalence ratio (CPR). Backward selection at a *p*-value of <0.1 was used to select variables for inclusion in the multivariable analysis. Before the multivariable regression model, we assess multicollinearity using the Variance Inflation Factor (VIF), resulting mean VIF of 4.10, suggesting no significant multicollinearity. Finally, a Modified Poisson regression model with robust variance estimator was fitted, adjusting for potential confounders including respondent age, to estimate the adjusted prevalence ratio (APR) with corresponding 95% Confidence intervals (CI). We used the Hosmer–Lemeshow test to assess the goodness-of-fit of our model [23]. Statistical significance was set at *p* < 0.05.

## Ethics and consent

The study is based on the publicly available 2022 TDHS datasets, which are accessible online and have been de-identified. The initial survey was approved by the National Institute of Medical Research Ethics Committee in Tanzania and the ICF Macro Ethics Committee in Calverton, New York. We obtained permission to use the DHS data from MEASURE Tanzania. The initial DHS was carried out in accordance with the principles of the Declaration of Helsinki. All methods were conducted following the relevant guidelines and regulations. The consent for publication is not applicable.

## Results

### Sociodemographic characteristics of study participants

A total of 3,246 men with a median age of 35 years (interquartile range = 29–42), with more than half (52.7%) aged 35–49 years. More than half (63.4%) had attained primary education, and 85.9% were literate. The majority (95.2%) were working, and 90.1% had media exposure. Regarding socioeconomic status, 36.0% of the participants were from poor households, while 43.7% were from rich households. Over half (61.0%) had ≤5 household members and 54.6% had ≤3 living children. One in five (20.6%) was alcohol consumers. More than half (69.0%) were from rural settings, and 30.0% were from the lake zone of mainland Tanzania (Table 2).

**Table 2.** Sociodemographic and behavioral characteristics of men in Tanzania (*N* = 3,246).

Characteristics	Frequency	Percentage
**Age group (years)**		
15–24	268	8.3
25–34	1,266	39.0
35–49	1,712	52.7
Median (IQR)	35 (29–42)	
**Education Level**		
No formal education	390	12.0
Primary	2,059	63.4
Secondary/Higher	798	24.6
**Marital Status**		
Married	2621	80.8
Cohabiting	316	9.7
Separated/Divorced	309	9.5
**Literacy**		
Illiterate	459	14.1
Literate	2,787	85.9
**Working Status**		
Not working	155	4.8
Working	3,091	95.2
**Media Exposure**		
No	322	9.9
Yes	2,924	90.1
**Number of living Children**		
None	215	6.6
1–3	1,771	54.6
≥4	1,260	38.8
Mean(±SD)	3.4 (2.6)	
**Alcohol Consumption**		
No	2,253	69.4
Yes	993	30.6
**Household members**		
≤5	1,980	61.0
≥6	1,266	39.0
**Spouse’s Currently Pregnant (*n* = 2,936)**		
No	2,548	86.8
Yes	388	13.2
**Wealth Index**		
Poor	1,168	36.0
Middle	660	20.3
Rich	1,417	43.7
**Sex of Household Head**		
Male	2,946	90.8
Female	300	9.2
**Own a mobile phone**		
No	402	12.4
Yes	2,844	87.6
**Internet use**		
Never use	2,359	72.7
Ever use	887	27.3
**Residence**		
Urban	1,007	31.0
Rural	2,239	69.0
**Geographical Zones**		
Western	273	8.4
Northern	348	10.7
Central	299	9.2
Southern	758	23.4
Lake	964	29.7
Eastern	508	15.7
Zanzibar	95	2.9

## Prevalence of men’s attitude toward justification of wife-beating

**Figure 1.**
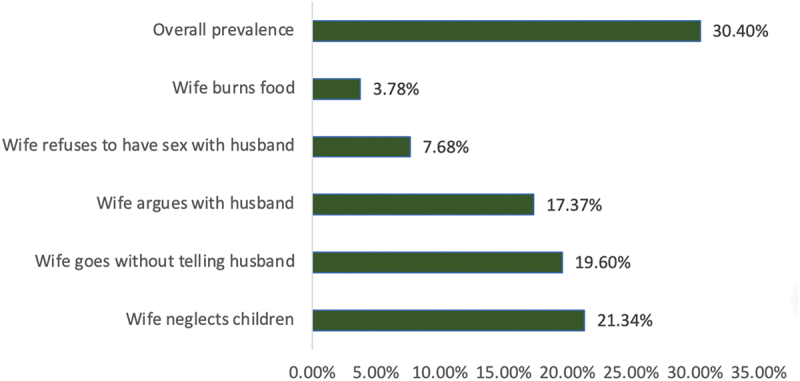
Percentage distribution of men’s attitudes toward justification of wife-beating in Tanzania.

The overall prevalence of men’s attitudes justifying wife-beating was 30.4%. Across specific scenarios, 21.34% of the men agreed that a wife deserves to be beaten if she neglects the children, whereas 3.78% justified wife-beating if she burns the food (Figure 1).

## Bivariate analysis of factors associated with men’s justification of wife-beating in Tanzania

Table 3 presents the bivariate analysis of the justification of wife-beating among the respondents’ characteristics. Respondent age, marital status, education, working status, alcohol consumption, sex of household head, mobile phone ownership, internet use, household wealth index, residence, and geographical zones were significantly associated with the likelihood of justification of wife-beating (*p* < 0.05). Contrarily, literacy, media exposure, number of living children, household members, and spouse’s pregnant status were not significantly associated with justification of wife-beating.

**Table 3.** The distribution of sociodemographic characteristics by men’s justification of intimate partner’s violence in Tanzania (*N* = 3246).

Characteristics	Justified wife-beating, *n* (%)		χ^2^ (df)	*p*-value
	No	Yes		
**Age group (years)**			28.1 (3)	0.001*
15–24	150 (56.1)	118 (43.9)		
25–34	873 (68.9)	393 (31.1)		
35–49	1,235 (72.2)	477 (27.8)		
**Education Level**			23.4 (2)	<0.001*
No formal education	250 (64.1)	140 (35.9)		
Primary	1401 (68.1)	658 (31.9)		
Secondary/Higher	607 (76.1)	191 (23.9)		
**Marital Status**			16.2 (2)	0.004*
Married	1862 (71.0)	759 (29.0)		
Cohabiting	191 (60.4)	125 (39.6)		
Separated/Divorced	205 (66.5)	104 (33.5)		
**Literacy**			4.7 (1)	0.070
Illiterate	299 (65.2)	160 (34.8)		
Literate	1959 (70.3)	828 (29.7)		
**Working Status**			5.7 (1)	0.039*
Not working	121 (78.3)	34 (21.7)		
Working	2137 (69.1)	954 (30.9)		
**Media Exposure**			2.0 (1)	0.256
No	213 (66.1)	109 (33.9)		
Yes	2045 (70.0)	879 (30.0)		
**Number of living Children**			4.3 (2)	0.225
None	137 (63.5)	78 (36.5)		
1–3	1232 (70.0)	539 (30.0)		
≥4	890 (70.6)	370 (29.4)		
**Alcohol Consumption**			32.5 (1)	<0.001*
No	1637 (72.7)	616 (27.3)		
Yes	621 (62.6)	372 (37.4)		
**Household members**			0.2 (1)	0.732
≤5	1372 (69.3)	608 (30.7)		
≥6	887 (70.0)	379 (30.0)		
**Spouse’s Currently Pregnant**				
No	1783 (70.0)	765 (30.0)	0.1 (1)	0.847
Yes	269 (69.3)	119 (30.7)		
**Wealth Index**			30.2 (2)	0.001*
Poor	743 (63.6)	425 (36.4)		
Middle	485 (73.5)	175 (26.5)		
Rich	1030 (72.7)	387 (27.3)		
**Sex of Household Head**			10.9 (1)	0.015*
Male	2075 (70.4)	871 (29.6)		
Female	183 (61.1)	117 (39.9)		
**Own a mobile phone**			13.4 (1)	0.003*
No	248 (61.6)	154 (38.4)		
Yes	2011 (70.7)	833 (29.3)		
**Internet Use**			24.9 (1)	0.001*
Never use	1482 (67.1)	777 (32.9)		
Ever use	676 (76.2)	211 (23.8)		
**Residence**			26.2 (1)	0.002*
Urban	763 (75.6)	244 (24.2)		
Rural	1495 (66.8)	744 (33.2)		
**Geographical Zones**			94.8 (6)	<0.001*
Western	185 (67.8)	88 (32.2)		
Northern	263 (75.7)	85 (24.3)		
Central	199 (64.5)	100 (83.5)		
Southern	527 (69.5)	231 (30.5)		
Lake	584 (60.6)	380 (39.4)		
Eastern	415 (81.7)	93 (18.3)		
Zanzibar	84 (88.7)	11 (11.3)		

χ^2^; Chi-square value, df; degree of freedom, *p < 0.05.

## Factors associated with men’s justification of wife-beating

Table 4 presents the adjusted prevalence ratios (APR) from the generalized Poisson regression analysis, revealing significant associations between several factors and the male justification of wife-beating. Compared to men aged ≥35 years, the prevalence of wife-beating justification was 60% higher among younger men aged 15–24 years (APR = 1.60, 95% CI: 1.31–1.96) and 16% higher among men aged 25–34 years (APR = 1.16, 95% CI: 1.01–1.34). Conversely, men with secondary or higher education were 27% less likely to justify wife-beating than those with no formal education (APR = 0.73, 95% CI: 0.57–0.94). Alcohol consumption was associated with a 33% increase in the prevalence of wife-beating justification (APR = 1.33, 95% CI: 1.16–1.52). Notably, men residing in western (APR = 2.25, 95% CI: 1.53–3.32), northern (APR = 1.77, 95% CI: 1.19–2.64), central (APR = 2.39, 95% CI: 1.63–3.51), southern (APR = 2.07, 95% CI: 1.46–2.91), and lake zones of mainland Tanzania (APR = 2.67, 95% CI: 1.89–3.80) were more likely to justify wife-beating compared to men in Zanzibar (Table 4).

**Table 4.** Generalized Poisson regression analysis for factors associated with justification of wife-beating among men in Tanzania (*N* = 3,246).

Characteristics	Unadjusted	*p*-value	Adjusted	*p*-value
	**PR (95%CI)**		**PR (95%CI)**	
**Age group (years)**				
15–24	1.57 (1.22–2.01)	<0.001*	1.60 (1.31–1.96)	<0.001*
25–34	1.12 (0.97–1.29)	0.134	1.16 (1.01–1.34)	0.042*
35–49	Ref		Ref	
**Education Level**				
No formal education	Ref		Ref	
Primary	0.89 (0.74–1.06)	0.198	0.90 (0.75–1.08)	0.278
Secondary/Higher	0.67 (0.53–0.84)	0.001*	0.73 (0.57–0.94)	0.013*
**Marital Status**				
Married	Ref		Ref	
Cohabiting	1.36 (1.15–1.62)	<0.001*	1.29 (1.09–1.53)	0.002*
Separated/Divorced	1.16 (0.93–1.43)	0.183	1.17 (0.95–1.45)	0.150
**Working Status**				
Not working	Ref		Ref	
Working	1.42 (0.99–2.03)	0.056	1.31 (0.91–1.87)	0.141
**Alcohol Consumption**				
No	Ref		Ref	
Yes	1.37 (1.20–1.57)	<0.001*	1.33 (1.16–1.52)	<0.001*
**Spouse’s Currently Pregnant**			-	
No	Ref			
Yes	1.02 (0.83–1.26)	0.842		
**Wealth Index**				
Poor	1.33 (1.14–1.55)	<0.001*	0.99 (0.83–1.99)	0.991
Middle	0.97 (0.79–1.17)	0.747	0.81 (0.66–0.99)	0.040*
Rich	Ref		Ref	
**Sex of Household Head**				
Male	Ref		Ref	
Female	1.31 (1.08–1.60)	<0.001*	1.16 (0.95–1.41)	0.140
**Residence**				
Urban	Ref		Ref	
Rural	1.37 (1.15–1.63)	<0.001*	1.17 (0.95–1.43)	0.134
**Geographical Zones**				
Western	2.84 (1.95–4.13)	<0.001*	2.25 (1.53–3.32)	<0.001*
Northern	2.14 (1.44–3.18)	<0.001*	1.77 (1.19–2.64)	0.005*
Central	2.95 (2.04–4.27)	<0.001*	2.39 (1.63–3.51)	<0.001*
Southern	2.69 (1.93–3.74)	<0.001*	2.07 (1.46–2.91)	<0.001*
Lake	3.47 (2.48–4.85)	<0.001*	2.67 (1.89–3.80)	<0.001*
Eastern	1.61 (1.07–2.42)	0.002*	1.47 (0.97–2.21)	0.067
Zanzibar	Ref		Ref	

PR; Prevalence ratio, CI: Confidence Intervals, Ref; Reference category, *p < 0.05.

## Discussion

This study examined the factors associated with male justification of wife-beating in Tanzania using data from the 2022 TDHS. The findings reveal that nearly one-third of Tanzanian men justify wife-beating under certain circumstances, highlighting a persistent societal acceptance of gender-based violence. This prevalence aligns with prior studies, where patriarchal norms and traditional gender roles normalize violence against women [24–26]. The Tanzanian wife-beating prevalence findings become particularly noteworthy when considering that women themselves often justify such violence in similar cultural contexts. For instance, in Nigeria, 62.4% of the women justified wife-beating in 2003, declining to 37.1% in 2013 [27]. This parallel suggests that wife-beating justification is not merely a male-perpetrated issue but a deeply internalized social norm affecting all genders. The Tanzanian figures likely reflect similar underlying patriarchal structures where violence becomes normalized through the generational transmission of gender norms [28]. The phenomenon of women justifying against themselves is observed across SSA [29] and underscores how profoundly patriarchal norms become embedded in collective consciousness. This context is important for interpreting why 30.4% of the Tanzanian men still justify wife-beating. When societies socialize both men and women to view gender-based violence as acceptable, challenging these attitudes requires transforming entire belief systems rather than simply targeting male perpetrators.

The study identified significant associations between wife-beating justification and sociodemographic factors, including age, education, marital status, alcohol consumption, and geographical zone of residence. Younger men (15–24 years) were significantly more likely to justify wife-beating compared to older men (≥35 years). The results align with research indicating that younger men may exhibit higher wife-beating justification due to limited exposure to gender-equitable norms and higher impulsivity [30,31]. Interventions targeting youth through education and awareness campaigns could help shift these attitudes.

Men with secondary or higher education were less likely to justify wife-beating than those without formal education. This finding is consistent with global evidence that education fosters gender-equitable attitudes and reduces acceptance of violence [32,33]. Additionally, alcohol consumption was strongly associated with wife-beating justification, corroborating previous studies that link alcohol use to increased aggression [34]. Alcohol may impair judgment and reinforce traditional masculine norms that condone dominance over women [35]. Public health interventions targeting alcohol abuse could contribute to reducing the acceptance of wife-beating.

Men in mainland Tanzania (Western, Northern, Central, Southern, and Lake zones) were more likely to justify wife-beating than those in Zanzibar. Regional disparities may stem from differences in cultural norms, enforcement of gender policies, or exposure to awareness programs [36]. Zanzibar’s lower prevalence of men’s justification of wife-beating could reflect stronger community-based interventions or distinct sociocultural dynamics. Future research should explore these regional differences, including involving a sufficient sample size to inform targeted interventions.

These results collectively demonstrate that wife-beating justification in Tanzania is not an isolated phenomenon but rather a manifestation of systemic gender inequalities that require multifaceted, context-specific solutions.

## Strength and limitations

The use of the 2022 TDHS provides a large, nationally representative dataset. This enhances the generalizability of the findings to the Tanzanian male population. Additionally, the findings might have direct implications for the development of evidence-based violence prevention programs and policies in Tanzania. The study addresses a critical gap in the literature by focusing on men’s justification of wife-beating, rather than solely focusing on women’s experiences as victims. This provides valuable insights into the perpetuation of wife-beating in Tanzania. The study utilizes a well-established and rigorous methodology, including a two-stage, stratified sampling design and Modified Poisson regression analysis with a robust variance estimator. This strengthens the validity and reliability of the findings. While providing valuable insights, this study’s findings should be interpreted cautiously due to its limitations. The cross-sectional design prevents causal inference, and reliance on self-reported data may introduce recall and social desirability biases. The use of the word ‘wife-beating’ during assessment could bring limited information, especially when the perpetrator is also doing the same violence to a girlfriend or any cohabiting female.

## Implications for practice and policy recommendations

This study’s findings suggest immediate and targeted interventions to disrupt the alarming prevalence of male justification of wife-beating in Tanzania. To effectively challenge harmful norms, we recommend developing and implementing evidence-based wife-beating prevention programs specifically for high-risk groups, including younger men, alcohol consumers, and cohabiting men, focusing on fostering respectful relationships and challenging harmful gender norms. Geographically targeted interventions should be prioritized in the Western, Northern, Central, Southern, and Lake Zones, addressing region-specific drivers of wife-beating justification. Integrating comprehensive gender equality and wife-beating prevention education into secondary and higher education curricula would be crucial for shaping positive attitudes from an early age. Strategic, multi-platform public awareness campaigns leveraging mass media could be essential to dismantling harmful gender norms and promoting respectful relationships. Legal frameworks must be reinforced to protect women from wife-beating, ensuring accountability for the perpetrators and promoting gender equality. In-depth qualitative studies should be conducted to understand the contextual drivers of wife-beating justification, and the effectiveness of existing prevention programs should be rigorously evaluated to inform evidence-based strategies.

## Conclusion

This study provides critical insights into the prevalence and determinants of male justification of wife-beating in Tanzania, revealing that a significant 30.4% of the men endorse beliefs that condone violence against women under specific circumstances. Notably, younger age, alcohol consumption, cohabitation, and residence in specific geographic zones were significantly associated with higher rates of wife-beating justification, while higher education demonstrated a protective effect. These findings underscore the urgent need for targeted interventions to address the harmful attitudes and beliefs that perpetuate wife-beating within the Tanzanian context. The identification of high-risk groups and geographic disparities highlights the importance of tailored prevention programs that challenge harmful gender norms and promote respectful relationships. Furthermore, the protective effect of higher education emphasizes the potential of educational interventions to foster positive attitudes and behaviors. Ultimately, this study calls for a multi-faceted approach, encompassing educational initiatives engaging young men, community engagement, and strengthened legal frameworks, to effectively combat wife-beating and promote gender equality in Tanzania.

## Supplementary Material

STROBE checklist Male Justification of IPV.docx

## Data Availability

The raw data supporting the conclusions of this article will be made available by the authors without undue reservation. The complete dataset is available at https://dhsprogram.com.
